# Pilot Phase II Trial of Bevacizumab Monotherapy in Nonmetastatic Castrate-Resistant Prostate Cancer

**DOI:** 10.5402/2012/242850

**Published:** 2012-06-13

**Authors:** Shin Ogita, Sheela Tejwani, Lance Heilbrun, Joseph Fontana, Elisabeth Heath, Stacy Freeman, Daryn Smith, Karen Baranowski, Ulka Vaishampayan

**Affiliations:** ^1^Department of Oncology, Wayne State University and Karmanos Cancer Center, Detroit, MI 48201, USA; ^2^Division of Hematology/Oncology, Henry Ford Health System, Detroit, MI 48202, USA; ^3^Department of Medicine, Section of Oncology, John D. Dingell VA Medical Center, Detroit, MI 48201, USA

## Abstract

*Introduction/Background*. Nonmetastatic castrate resistant prostate cancer (CRPC) is a challenging disease state. The objective of this study was to evaluate the efficacy and tolerability of bevacizumab in nonmetastatic CRPC patients. *Patients*. Patients with prostate cancer who developed PSA recurrence after local therapy were included if they had absence of bone or visceral metastases and PSA progression despite androgen deprivation therapy. *Methods*. Bevacizumab 10 mg/kg intravenously was administered every 14 days until PSA progression, development of metastasis, or unacceptable toxicity. *Results*. 15 patients were enrolled and treated with bevacizumab for a median duration of 3.1 months. Median baseline PSA was 27 ng/mL, and seven patients had Gleason Score ≥8. Five patients had declined in PSA during the treatment. Median PSA doubling time was prolonged from 4.7 months pretreatment to 6.5 months. Median time to PSA progression and new metastasis were 2.8 and 7.9 months, respectively. There were three grade 3 adverse events (one proteinuria and two hypertension) and one pulmonary embolism. There was no treatment-related mortality. *Conclusion*. Bevacizumab therapy had minimal impact on the disease course of nonmetastatic CRPC, and investigation of novel strategies is needed.

## 1. Background

Approximately 30–40% of localized prostate-cancer patients develop biochemical relapse at 10 years after definitive local treatments [1–3]. Case series reports indicate that patients with prostate specific antigen (PSA) relapse will develop metastatic disease within a median duration of eight years, after biochemical failure [4]. Patients with high Gleason score (≥8), rapid PSA doubling time, and/or earlier PSA relapse after local treatment have higher risk of progression to metastatic disease during their lifespan and higher mortality [4, 5]. 

Currently no systemic therapy has proven efficacy in delaying the appearance of metastatic disease or improving survival after biochemical relapse (PSA failure). LHRH agonists are the most widely used agents in this setting. Although there is no solid evidence to support this strategy, randomized trials in metastatic disease certainly suggest that immediate use of androgen deprivation therapy (ADT) is associated with improved disease-specific mortality and morbidity, compared with delayed initiation of the therapy [6]. Unfortunately, progression on ADT eventually occurs, that is, nonmetastatic castrate-resistant prostate cancer (CRPC), and after that, this patient population is likely to continue progression to metastatic disease. Prospective studies have shown that higher baseline PSA and higher PSA velocity (i.e., shorter PSA doubling time) are the independent predictors for shorter time to bone metastasis and worse overall survival [7, 8]. A recently reported phase III study showed that denosumab, a RANKL monoclonal antibody, delayed time to bone metastasis by 3.7 months when compared to placebo in nonmetastatic CRPC patients; however the impact on morbidity or overall survival is unknown [9]. Therefore, currently there remains an unmet need for evaluation of effective and tolerable agents in the therapy of non-metastatic CRPC to intervene and alter outcomes, prior to the appearance of distant metastases.

Bevacizumab is a fully humanized anti-VEGF monoclonal IgG_1_ antibody [10]. In phase II studies, the addition of bevacizumab to docetaxel demonstrated 37–49% objective response rates in patients who had been pretreated with docetaxel [11, 12]. The addition of bevacizumab appeared to be safe and did not increase serious adverse events in prostate cancer patients in both metastatic and nonmetastatic settings [11–13]. With the preliminary efficacy data in metastatic prostate cancer and the established tolerability of bevacizumab, it led to the rationale of evaluating the agent in an earlier setting of nonmetastatic disease. The objectives of this phase II study were to evaluate the efficacy and tolerability of bevacizumab monotherapy in non-metastatic prostate cancer patients, with biochemical progression, after local therapy and ADT.

## 2. Patients

Patients 18 years of age or older with an Eastern Cooperative Oncology Group (ECOG) performance status of either 0 or 1 and life expectancy of at least 6 months were eligible if they had a documented histological diagnosis of prostate adenocarcinoma, with no evidence of bone/visceral metastases, as visualized on standard imaging such as bone scan, chest X-ray, CT scan, or MRI of abdomen and pelvis. Patients had to have biochemical progression by prostate-specific antigen (PSA) levels, despite being treated with androgen deprivation therapy. PSA progression was defined as 3 rising levels, with a minimum interval of 2 weeks between each determination. The last PSA level had to be a minimum value of 1 ng/mL, measured within two weeks prior to registration. If the patient was on antiandrogen therapy, then PSA progression after the withdrawal period (a minimum of 28 days for flutamide and 42 days for bicalutamide or nilutamide) was required. Patients with a history of previous exposure to bevacizumab were excluded and no concurrent anticancer therapies including antiandrogen therapy and other hormonal manipulations, except for LHRH agonists were permitted during the study. Patients on steroids had to discontinue them before starting bevacizumab. Patients who were on LHRH agonists were required to continue the use of this therapy. Bisphosphonate use was allowed per treating physician discretion. At least 4 weeks had to have elapsed since prior systemic therapy, except for LHRH analogue therapy and steroids. Patients were required to use effective means of contraception. Patients with uncontrolled hypertension (defined as systolic blood pressure >150 and/or diastolic blood pressure >100 mmHg on antihypertensive medications), any prior history of hypertensive crisis or hypertensive encephalopathy, New York Heart Association (NYHA) grade 2 or greater congestive heart failure, history of myocardial infarction or unstable angina within 12 months from the study enrollment, and/or history of stroke or transient ischemic attack within 6 months were excluded. Patients with significant vascular disease (e.g., aortic aneurysm, aortic dissection), symptomatic peripheral vascular disease, evidence of bleeding diathesis or coagulopathy, history of abdominal fistula, gastrointestinal perforation, or intra-abdominal abscess within 6 months prior to study enrollment were also ineligible. Patients on anticoagulants were allowed if they had been on therapy for at least 4 weeks and had no acute thromboembolic activity. Patients had to have recovered from any major surgical procedure, open biopsy, or significant traumatic injury without serious, nonhealing wound, ulcer, or bone fracture, and a minimum time interval of 28 days must have elapsed from any major surgery. Patients with significant proteinuria (urine protein: creatinine ratio ≥1.0) were excluded. Low-dose aspirin (≤325 mg/d) could be continued in subjects at high risk for arterial thromboembolic disease.

All the patients reviewed and signed written informed consent, and the protocol was approved by the institutional review board of each participating hospital.

## 3. Treatment

Bevacizumab monotherapy was administered on an outpatient basis at a dose of 10 mg/kg intravenously every 14 days. The treatment was continued until disease progression, intolerable toxicities, or patient withdrawal from the study. Dose reduction of bevacizumab was not allowed. If patients experienced any of the following adverse events (AEs), bevacizumab was discontinued permanently and the patient was taken off the study: grade 3 hypertension not controlled by antihypertensive medications, grade 4 hypertension, any grade 4 hemorrhage, grade ≥2 pulmonary and/or cranial hemorrhage, symptomatic grade 4 venous thrombotic event, arterial thromboembolic event of any grade, grade 4 congestive heart failure, grade 4 proteinuria, gastrointestinal perforation, wound dehiscence requiring interventions, and any grade 4 events thought to be related to bevacizumab by investigators. The treatment was delayed for the following AEs: grade 3 nonpulmonary and/or non-CNS hemorrhage, grade 3 venous thrombosis, grade 3 proteinuria, grade ≥2 bowel obstruction, and any grade 3 events. Bevacizumab was withheld at least 2 weeks prior to any minor procedure (e.g., dental extraction, superficial skin lipoma removal) and at least 4 weeks prior to any major surgical procedure. The therapy was not restarted for a minimum of 4 weeks after a major surgical procedure and until wound healing was complete. Regardless of the reason for holding study drug treatment, the maximum allowable length of treatment interruption was 2 months.

## 4. Evaluation

Blood pressure and proteinuria were monitored every two weeks and 4 weeks, respectively. Toxicity was evaluated every 2 weeks and PSA evaluated every 6 weeks while on therapy. PSA was evaluated after discontinuing therapy at a minimum of every 3 months. Bone scan, CXR or chest CT, and CT scan of abdomen and pelvis were performed every 3 months while on therapy, and after that at physician discretion. Toxicities were graded per the National Cancer Institute Common Terminology Criteria for Adverse Events (CTCAE) version 3.0, and PSA response was determined per the PSA working group criteria [14]. A PSA decline ≥50%, confirmed by a second PSA value at least 4 weeks later, was considered a PSA response if patients did not demonstrate clinical or radiographic evidence of disease progression during this time period. The reference PSA for these declines was a PSA measured within 2 weeks before starting therapy. PSA progression was defined differently for patients who demonstrated a PSA response versus not. In patients whose PSA had not decreased, progression was defined as a 25% increase over the baseline (before study) and an increase in the absolute value PSA level by at least 1 ng/mL, which was confirmed by a second value at least 4 weeks later. In patients whose PSA had decreased, progressive disease was considered to have occurred when PSA increased 25% over the nadir, provided that the increase was a minimum of 1 ng/mL and was confirmed by a second value at least 4 weeks later. In patients whose PSA declined to 50% of baseline on study, PSA progression was defined as a 50% increase over the nadir and a minimum of 1 ng/mL increase, confirmed by a second value at least 4 weeks later.

## 5. Statistical Methods

This single arm multi-institutional phase II trial was a pilot study. Primary endpoints of this study were PSA response rate, time to PSA progression (TTPP), and treatment-related toxicities. TTPP was measured from the registration date to the date of PSA progression. Secondary endpoints included overall survival (OS) and time to metastatic disease which was measured as the time from registration to the first clinical or radiologic appearance of metastases. The sample size of 15 was planned to estimate the PSA response rate (or toxicity rates) with sufficient precision such that the estimate will be useful in planning a subsequent study. With *N* = 15, the PSA response proportion (or a toxicity rate) could be estimated with a standard error <0.13. 

Statistical graphics were used to display the PSA response distribution (via a waterfall plot), and the statistics of the PSA distributions at various time points (via a multiple box plot). Standard Kaplan-Meier estimates of the censored TTPP, time to metastatic disease, and time to bone metastasis distributions were computed. Due to the small sample sizes, time to event statistics (e.g., median, 6 month rate, etc.) were estimated more conservatively using linear interpolation among successive event times on the Kaplan-Meier curves [15].

## 6. Results

### 6.1. Patient Characteristics

A total of 16 patients were enrolled in the study from December, 2007 to November, 2010 in three hospitals in Detroit, Michigan in the United States. However, one patient was never treated due to ineligibility (uncontrolled hypertension) and excluded from this analysis. Therefore, the results of the 15 eligible and treated patients are reported here.

The baseline characteristics of the study participants are summarized in Table 1. Median age was 70 years (range 51–87 years) and 4 patients were over 80 years of age. None of the patients had prior chemotherapy, and fourteen patients had prior radiation as a local treatment. Seven patients had a Gleason score of 8 or higher at diagnosis. Two patients had PSA recurrence within two years following the completion of their local therapy. The baseline PSA doubling time was 4.7 months before therapy, and all the patients had PSA doubling time ≤10 months, which are associated with poor prognosis. Median PSA prior to the initiation of bevacizumab was 27 ng/mL (range 2.6–104.0), and ten patients had baseline PSA ≥10 ng/mL.

### 6.2. Toxicity

Fourteen patients had discontinued bevacizumab by the time of this analysis. Thirteen patients discontinued the therapy due to disease progression, and one patient discontinued bevacizumab due to occurrence of grade 4 pulmonary embolism, which occurred during the third cycle of treatment. No treatment-related mortality was observed. All other treatment-related AE of grade 2 or worse are summarized in Table 2. The most common AE was hypertension. Two patients developed grade 3 hypertension requiring dose adjustment of their antihypertensive drugs.

### 6.3. Treatment Efficacy and Survival

**Figure 1:**
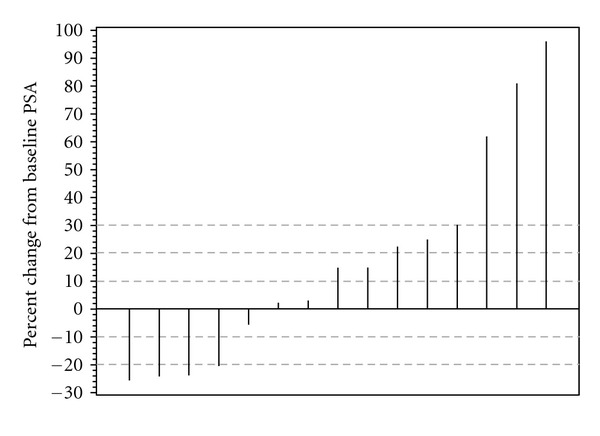
Waterfall plot of the PSA response distribution.

**Figure 2:**
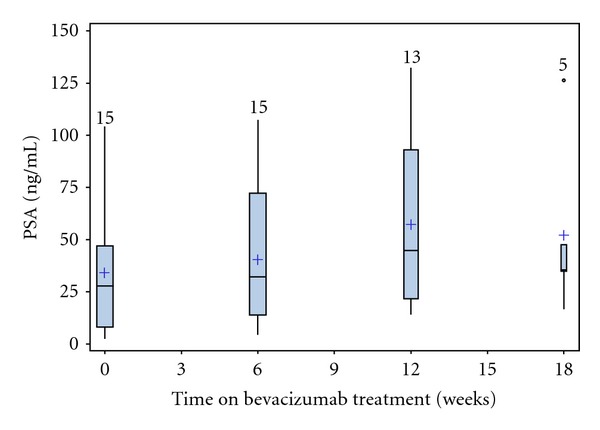
Multiple box plot of the statistics of the PSA distributions at various time points. The vertical lines represent range of PSA with boxes indicating interquartile range. The horizontal lines in the boxes represent median PSA and “+” represents mean value. The numbers above the vertical lines are the number of patients. The circle of week 18 represents one patient whose PSA value was more than twice the interquartile range.

Median treatment duration was 3.1 months (range 2.6–12.1 months). PSA decline was observed in 5 patients, but all of them were <50% reduction and did not meet the criteria for partial response.  Figure 1 describes the best PSA response of each individual patient on study in a waterfall plot. The summary statistics of the distribution of PSA change are shown in Figure 2 by a multiple box plot. Median PSA doubling time slowed from 4.7 months at baseline to 6.5 months during the treatment with bevacizumab (Table 3).

**Figure 3:**
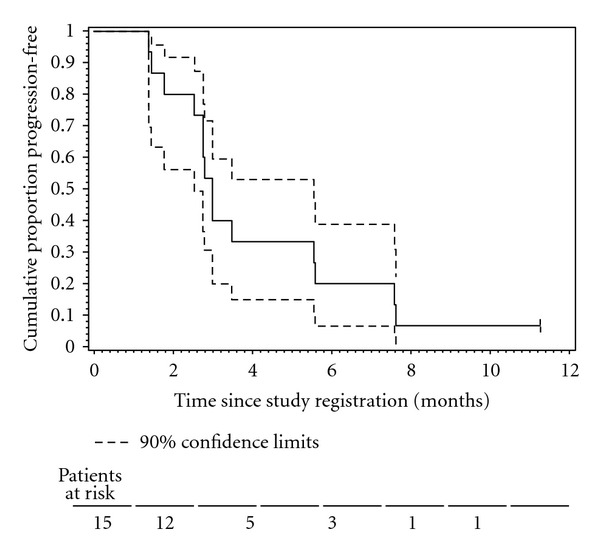
The Kaplan-Meier plot of the censored time to PSA progression (TTPP) distribution.

**Figure 4:**
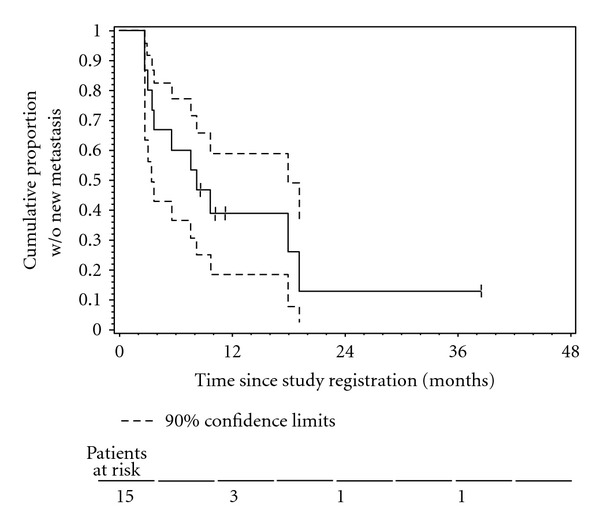
The Kaplan-Meier plot of the censored time to new metastasis distribution.

Median time to PSA progression (TTPP) was 2.8 months (90% CI 2.4–5.4 months), with six month TTPP rate of 19% (90% CI 2–36%). The Kaplan-Meier curve of TTPP is shown in Figure 3. Median time to new metastasis was 7.9 months (90% CI 3.2–17.6 months), with 6-month and 12-month metastasis-free survival rates of 59% and 35%, respectively (Figure 4). Median time to bone metastasis was 10.6 months.

Only two patients have died to date; therefore it is premature to analyze the OS data. Both deaths were related to disease progression.

## 7. Discussion

Angiogenesis is one of the key mechanisms of tumor growth and survival. Increased vascular density has been observed in prostate cancer versus that noted in normal prostate tissue [16]. Increased vascularity in the primary prostate tumor was found to be associated with increased risk of disease metastasis [17]. In mice xenograft models, bevacizumab monotherapy showed suppression of tumor growth and prevention of metastasis [18]. Phase II trials revealed promising efficacy of bevacizumab containing therapy in metastatic CRPC who had been previously heavily treated with chemotherapies with PSA response rates of 55–63%, objective response rates of 37–49%, and median survival of 9 months or longer [11, 12]. However, recently reported results of the phase III trial in metastatic CRPC revealed that the addition of bevacizumab to standard docetaxel and prednisone regimen improved PFS and response rate but failed to demonstrate overall survival benefit and increased treatment-related deaths [19].

In our study, median TTPP and median time to new metastasis were 2.8 and 7.9 months, respectively. These estimated medians have wide confidence intervals due to the small sample size, but also reflect the heterogeneity of this disease state which has made conducting clinical trials extremely difficult. Although the median PSA doubling time was increased from 4.7 months before therapy to 6.5 months during bevacizumab, it is unknown if this change was directly related to the bevacizumab use or not. Moreover, the clinical significance of this change is uncertain, and the prolongation of PSA doubling time has not been clearly associated with better clinical outcomes in nonmetastatic CRPC patients.

Our TTPP outcome time points and median time to bone metastases were relatively short compared to other clinical studies in this disease state and to historical data [8, 9, 20]. There are several possible explanations for this outcome. First of all, our patients had multiple poor prognostic features. All the patients had PSA doubling time of less than 10 months, and half of the patients had Gleason Score ≥ 8. Secondly, our patient population had a higher proportion of patients with locally advanced disease stage at diagnosis, which explains why most patients had received radiation therapy as the primary local therapy. Third, bevacizumab may not be effective enough to alter clinical outcomes in the micrometastatic setting, in prostate cancer, especially given the lack of OS benefit in metastatic CRPC from the recently published results of a phase III clinical trial [19]. One of the mechanisms of action of bevacizumab is through its effects on stroma without direct cytotoxic effect [21]. A perfect example of a similar phenomenon is that, in colon cancer, the addition of bevacizumab to cytotoxic chemotherapy improved overall survival in metastatic disease, but not in the adjuvant setting [22, 23]. Moreover, in a preclinical model, bevacizumab was found to be less active against CRPC than hormone-sensitive prostate cancer [24].

Nonmetastatic CRPC remains a challenging disease. So far, several agents have been tested without successful outcomes [20, 25, 26]. A large randomized phase III study treated nonmetastatic CRPC patients with atrasentan, an oral selective endothelin-A receptor antagonist, or placebo [20]. Atrasentan improved time to disease progression (similar definition to the time to new metastasis in our study) from 22.4 months to 25.5 months, but the difference was not statistically significant (*P* = 0.288). The atrasentan study also illustrated the different practice patterns by regions. Forty percent of US patients discontinued the treatment prematurely primarily due to rising PSA whereas only 21.9% of the patients in other countries did so. Some patients and/or physicians may feel uncomfortable with continuing the treatment knowing the rising PSA. We designed our study protocol which would discontinue bevacizumab upon PSA progression conforming to real practice in the USA. This may have been a flawed assessment since the impact of bevacizumab monotherapy on PSA levels is unknown.

Another small randomized phase II study compared pox-virus-based PSA vaccine with the antiandrogen, nilutamide in nonmetastatic CRPC patients [26]. Possible survival benefit was observed in those who received initial immunotherapy, and a larger scale study is currently ongoing [27].

Denosumab is a monoclonal antibody against RANKL and inhibits the osteoclast activity. Denosumab demonstrated a delay in progression to bone metastasis in high-risk nonmetastatic CRPC patients, which were defined as PSA ≥ 8 ug/L and/or PSA doubling time ≤10 months [9]. However, this change did not translate into an overall survival benefit. Furthermore, risk of osteonecrosis of jaw was 5% and the quality-of-life impact of this adverse event on asymptomatic patient, needs to be seriously considered.

Studying nonmetastatic CRPC is extremely difficult due to the heterogeneity of the population, the unpredictable natural disease course, and the lack of established endpoints to assess efficacy of novel agents in this setting. The definition of “nonmetastatic” prostate cancer continues to change with wider application of more sophisticated imaging techniques such as magnetic resonance imaging (MRI) and positron emission tomography (PET) scan [28]. Incidentally found metastases are now detected in as many as 30% of asymptomatic CRPC patients, which makes study screening failure rate extremely high and causes slower accrual [29]. Including specific high-risk subsets, based on Gleason Score or PSA doubling time criteria, may help limit the heterogeneity in the study population. Future studies will need to consider stringent eligibility criteria.

The additional challenge in this population is that patients are asymptomatic and subjecting them to potentially toxic therapies requires careful consideration. Bevacizumab is generally considered a well-tolerated biologic agent, but it can potentially cause significant side effects, and 4 out of 15 patients in our study developed grade 3 or higher adverse events. However, if safe and effective treatment becomes available, then the non-metastatic disease state represents an ideal setting for intervention during this window of opportunity.

Designing good clinical studies for nonmetastatic prostate cancer is a dire need, and meeting the challenge of clinical trial accrual in this asymptomatic but threatened patient population should remain a priority.

## 8. Conclusion

In summary, bevacizumab monotherapy had only a modest impact at best, on PSA doubling time and no clinical impact in nonmetastatic CRPC patients. Further investigation of bevacizumab monotherapy in prostate cancer is not indicated.

## Figures and Tables

**Table 1:** Baseline characteristics.

	Patients (*N* = 15)
Median age (range)	70 (51–87)

Race	Caucasian 9 (60%) African American 6 (40%)

Performance status	Zero 9 (60%) One 6 (40%)

Prior local treatment	Prostatectomy 3 (20%); radiation 14 (93%); cryotherapy 1 (7%)

Other prior therapies	Antiandrogen 13 (86%) Ketoconazole 3 (20%) Steroid 2 (13%)

Gleason score	Six 4 (27%); seven 4 (27%), eight 1 (7%), nine 6 (40%)

Median pretreatment PSA [ng/mL] (range)	27 (2.6–104)
Median Hgb [g/dL] (range)	12.9 (9.9–15.5)

**Table 2:** Toxicities.

	Gr 2	Gr 3	Gr 4
GU bleeding	1 (6.6%)		
Thromboembolism			1 (6.6%)
Proteinuria	2 (13.3%)	1 (6.6%)	
HTN	7 (46.6%)	2 (13.3%)	
Diarrhea	1 (6.6%)		
Nausea/vomiting	1 (6.6%)		
Anemia	1 (6.6%)		

**Table 3:** PSA response.

	*N*	Median	Min	Max
PSA doubling time (months) pretreatment	15	4.7	0.8	9.9

PSA doubling time (moths) on therapy	15	6.5	0.8	29.4
